# Lack of Renoprotective Effect of Chronic Intravenous Angiotensin-(1-7) or Angiotensin-(2-10) in a Rat Model of Focal Segmental Glomerulosclerosis

**DOI:** 10.1371/journal.pone.0110083

**Published:** 2014-10-22

**Authors:** Juan Carlos Q. Velez, Michael G. Janech, Megan P. Hicks, Thomas A. Morinelli, Jessalyn Rodgers, Sally E. Self, John M. Arthur, Wayne R. Fitzgibbon

**Affiliations:** 1 Division of Nephrology, Department of Medicine, Medical University of South Carolina, Charleston, South Carolina, United States of America; 2 Medical Service, Ralph H. Johnson Veterans Affairs Medical Center, Charleston, South Carolina, United States of America; 3 Department of Pathology, Medical University of South Carolina, Charleston, South Carolina, United States of America; IRCSS - Istituto di Ricerche Farmacologiche Mario Negri, Italy

## Abstract

Unopposed angiotensin (Ang) II-mediated cellular effects may lead to progressive glomerulosclerosis. While Ang-II can be locally generated in the kidneys, we previously showed that glomerular podocytes primarily convert Ang-I, the precursor of Ang-II, to Ang-(1-7) and Ang-(2-10), peptides that have been independently implicated in biological actions opposing those of Ang-II. Therefore, we hypothesized that Ang-(1-7) and Ang-(2-10) could be renoprotective in the fawn-hooded hypertensive rat, a model of focal segmental glomerulosclerosis. We evaluated the ability of 8–12 week-long intravenous administration of either Ang-(1-7) or Ang-(2-10) (100–400 ng/kg/min) to reduce glomerular injury in uni-nephrectomized fawn-hooded hypertensive rats, early or late in the disease. Vehicle-treated rats developed hypertension and lesions of focal segmental glomerulosclerosis. No reduction in glomerular damage was observed, as measured by either 24-hour urinary protein excretion or histological examination of glomerulosclerosis, upon Ang-(1-7) or Ang-(2-10) administration, regardless of peptide dose or disease stage. On the contrary, when given at 400 ng/kg/min, both peptides induced a further increase in systolic blood pressure. Content of Ang peptides was measured by parallel reaction monitoring in kidneys harvested at sacrifice. Exogenous administration of Ang-(1-7) and Ang-(2-10) did not lead to a significant increase in their corresponding intrarenal levels. However, the relative abundance of Ang-(1-7) with respect to Ang-II was increased in kidney homogenates of Ang-(1-7)-treated rats. We conclude that chronic intravenous administration of Ang-(1-7) or Ang-(2-10) does not ameliorate glomerular damage in a rat model of focal segmental glomerulosclerosis and may induce a further rise in blood pressure, potentially aggravating glomerular injury.

## Introduction

Angiotensin (Ang) II has been implicated in the pathogenesis of various glomerular diseases, such as diabetic glomerulopathy, focal segmental glomerulosclerosis (FSGS), IgA nephropathy, and others [1]–[3]. Ang-II is primarily formed after the cleavage of Ang-I by Ang-converting enzyme (ACE). In the kidney glomerulus and other organs, Ang-I can also be converted to other Ang fragments by the action of various peptidases. Neprilysin converts Ang-I into the heptapeptide Ang-(1-7), whereas aminopeptidase A converts it into the nonapeptide Ang-(2-10) [4], [5]. Furthermore, Ang-(1-7) can also be generated by cleavage of Ang-II by ACE2, prolylcarboxypeptidase or prolylendopeptidase by other cell types that reside in the kidney [6], [7].

It has been recognized that Ang-(1-7) may exert cellular actions by stimulation of a specific receptor, the *mas* receptor [8], that are antagonistic to those of Ang-II, including a vasodilatory [9], natriuretic [10], antiproliferative [11] and antifibrotic effect [12]. Those observations led others to postulate that Ang-(1-7) could be a protective peptide in glomerular diseases. Indeed, chronic subcutaneous administration of Ang-(1-7) was shown to be protective in rodent models of diabetic glomerulopathy [13] and anti-Thy1.1 nephritis [14]. In contrast, others have found that Ang-(1-7) is not protective in models of progressive glomerulosclerosis [15] and FSGS [16], and is detrimental in models of diabetic glomerulopathy [17]. Of note, most laboratories studied early stages of the disease and administered the heptapeptide for only 1–6 weeks [18]. Therefore, we opted to expand the investigation of the effect of Ang-(1-7) to advanced stages of glomerular disease and during longer duration of treatment.

Previous work demonstrated that rat glomeruli primarily convert Ang-I to Ang-(1-7) and Ang-(2-10) [4]. Studies from a single laboratory suggested that Ang-(2-10) may modulate the pressor actions of Ang-II [19]. However, the effects of chronic systemic administration of Ang-(2-10) have not been studied to date. In addition, Ang-(2-10) could be converted to Ang-III by ACE, and Ang-III has been proposed to promote natriuresis by virtue of being the predominant agonist of tubular Ang-II type 2 (AT_2_) receptors [20]. Therefore, we also evaluated the effect of chronically infused Ang-(2-10) on kidney damage in a model of glomerular disease.

Thus, we hypothesized that Ang-(1-7) and/or Ang-(2-10) may ameliorate glomerular damage in a rat model of FSGS by diminishing the degree of Ang-II-mediated injury. To test our hypothesis, we selected the fawn-hooded hypertensive (FHH) rat, a well characterized model of spontaneous hypertension and proteinuria associated with a histological lesion of FSGS [21]–[23]. In addition, because stimulation of the Ang-II type 2 (AT_2_) receptor has been reported to counteract some of the detrimental effects of Ang-II via the AT_1_ receptor, we assessed the ability of Ang-(1-7) and Ang-(2-10) to bind to Ang receptors.

## Materials and Methods

### Ang peptide radioligand binding assay

HEK-293 cells stably expressing AT_1a_ receptor were maintained and characterized as previously described [24]. HEK-293 cells expressing AT_2_ receptors were transfected with pcDNA3.1 plasmid containing human AT_2_ receptor DNA using Lipofectamine 2000 at 1 µg DNA/well of a six-well plate according to manufacturer's protocol (Invitrogen, Carlsbad, CA). Transfected cells were maintained in Minimal Essential Medium (MEM) containing 10% fetal bovine serum and 1% antibiotic/fungizone/antimycotic with addition of geneticin (400 µg/ml) for stable transfectants. Confluent monolayers cells were deprived of serum (0.1% bovine serum albumin) for 2–24 hours before assay. Cells were placed on ice for 15 minutes followed by addition of assay buffer (50 mM Tris pH 7.4, 100 mM NaCl, 12 mM MgCl, 2 mM KCl, 1% BSA and freshly added bacitracin) or various concentrations of competing ligand and [^125^I]-Ang-II (∼200,000 cpm/well) in a total volume of 1 ml/well. After 90 min of incubation on ice, cells were gently washed 3 times with ice cold 0.9% sodium chloride followed by solubilization of the cell monolayer with 0.1 N NaOH/0.1% SDS. The solubilized sample was placed into a gamma counter for determination of cell-associated radioactivity. Log-logit analysis of the obtained competition curves was performed for determination of IC50 values.

### Animal Procedures

#### Ethics statement

All animal procedures were conducted with approval from the Medical University of South Carolina Institutional Animal Care and Use Committee and in accordance with the procedures and practices of the *NIH Guide for the Care and Use of Laboratory Animals*.

Male FHH rats (In-house colony established from animals obtained from Charles River Laboratories, Wilmington, MA) were housed at a certified animal facility at 22°C and a 12∶12 h light/dark cycle. The rats were allowed free access to tap water, fed a standard diet (Teklad #2918, Harlan Laboratories, Madison, FWI) and provided with environmental enrichment. All surgeries were performed under isoflurane anesthesia (5% induction, 2% maintenance), at 37°C, and perioperative buprenorphine.

### Model of FSGS

**Figure 1:**
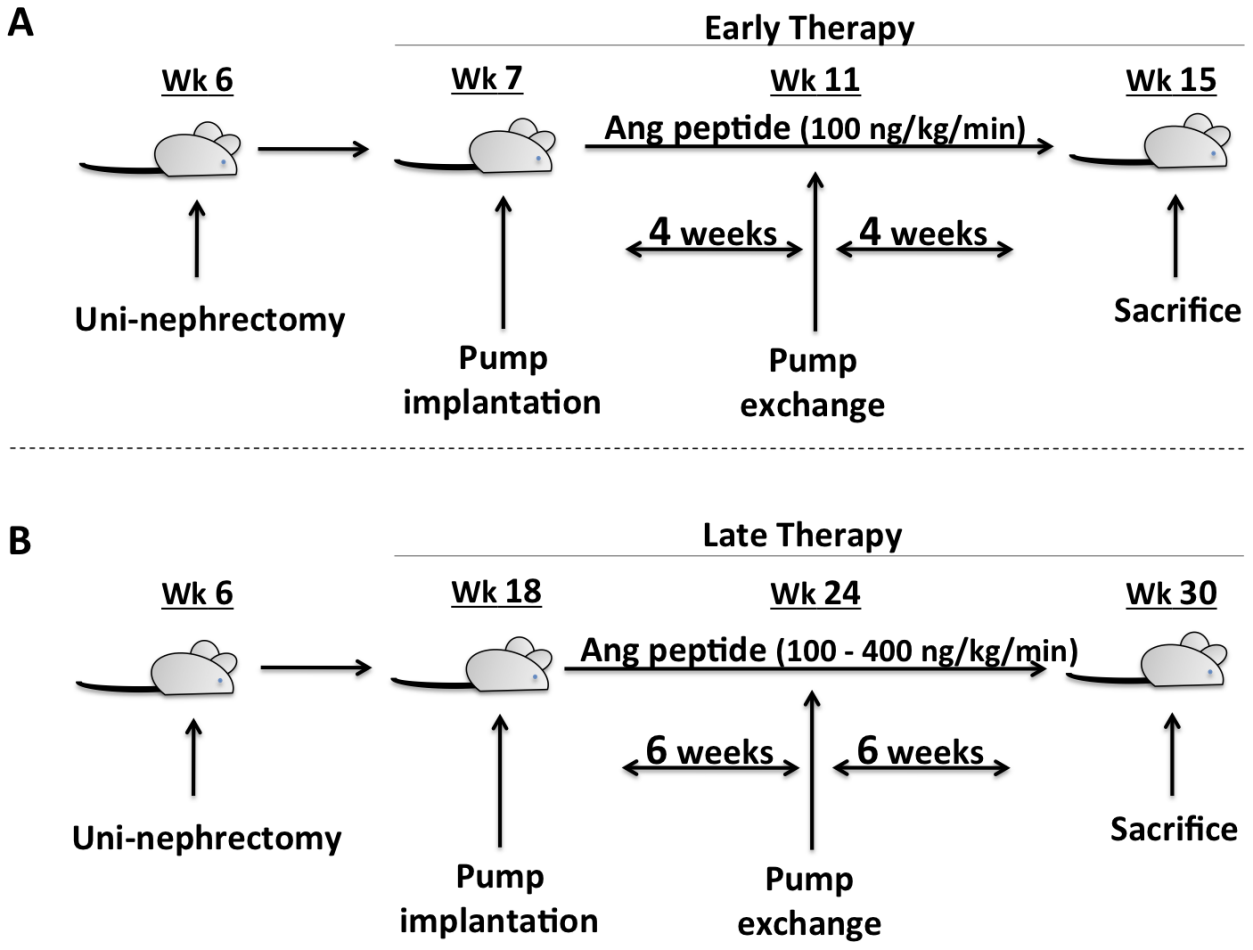
Experimental protocols. Schematic of treatment protocols employed to test the effect of early (Panel *A*) or late (Panel *B*) chronic intravenous administration of Ang-(1-7) and Ang-(2-10) on the progression of glomerulosclerosis in uni-nephrectomized FHH rats.

A schematic of the animal protocols is shown in Figure 1. In order to accelerate the development of glomerulosclerosis [25], rats (95–205 g) underwent left nephrectomy at 6 weeks of age. Following anesthesia, an incision was made in the left flank; the kidney was exposed and excised after ligation of the pedicle. One (early disease) or 12 (late disease) weeks following single nephrectomy, the rats underwent implantation of osmotic mini-pumps (Alzet models 2004 or 2006 Alza Corp., Palo Alto, CA) for intravenous delivery of either isotonic saline (vehicle) or Ang peptides. Under anesthesia, a mini-pump was placed in a subcutaneous pocket. A catheter (PE 50) attached to the pump was then tunneled subcutaneously around the neck and cannulated into the right jugular vein. Pumps were replaced once during the treatment period. For the early disease model, five groups of rats were examined. Either saline (n = 4) or 100 ng/kg/min (low-dose, LD) of Ang II (n = 4), Ang-(1-7) (n = 4) or Ang-(2-10) (n = 4) was infused for a total of 8 weeks. An additional group (n = 5) received captopril (100 mg/kg/d) in the drinking water throughout the treatment period. For the late disease model, eight groups of rats were examined. Saline (n = 7) or 100 ng/kg/min (low-dose, LD) or 400 ng/kg/min (high-dose, HD) of either Ang-(1-7) or Ang-(2-10), or Ang II (LD), was infused for a total of 12 weeks (n = 9 per Ang peptide treatment). The dosages of the Ang peptides were selected based on previous studies where Ang-(1-7) was similarly administered using implanted osmotic mini-pumps [13], [16], [17], [26]. Two further groups received captopril (100 mg/kg/d, n = 10) or losartan (20 mg/kg/d, n = 9) orally throughout the treatment period. Systemic blood pressure was measured by tail-cuff sphygmomanometer (Natume, Japan). Animals were placed on metabolic cages for urine collection at pre-specified time points. Urinary protein excretion was measured by the method of Lowry [27]. Urinary sodium concentration was measured by ion-selective electrodes. At sacrifice, kidneys were harvested for histological analysis and measurement of Ang peptide concentration.

### Histological Analysis

Upon harvesting, kidneys were cleared of blood by transcardiac perfusion of iced-cold phosphate buffered saline, excised and fixed in 4% paraformaldehyde, embedded in paraffin and processed for microscopic examination. Kidney sections (2-µm) were stained with hematoxylin-eosin (H&E), Jones silver and Periodic Acid Schiff (PAS). Slides were reviewed by an experienced renal pathologist (S.S) in a blinded fashion. Index of glomerulosclerosis was evaluated by a semiquantitative score based on the percentage of the glomerular tuft occupied by sclerotic lesions (0: no lesions, 1: lesions affecting less than 25% of the glomerulus, 2-3-4: lesions affecting 25–50%, 50–75% and 75–100% of the glomerulus, respectively). Tubular injury was rated based on presence of tubular atrophy, microcystic tubular dilation and interstitial fibrosis, from 0 to 3 (0: none; 1: mild; 2: moderate; 3: severe).

### Ang Peptide Content Determination

Stable isotope labeled peptide standards (SIS, ^13^C ^15^N, New England Peptide, Gardner, MA) were resuspended in water to 1 nM and stored at −80C in Lo-Bind microcentrifuge tubes (Eppendorf) with the exception of Ang-I, which was resuspended in 30% acetonitrile and stored at 0.4 nM final concentration. Ang-(1-7), Ang-II, and Ang-(2-10) contained isotopically labeled valine. Ang-I contained isotopically labeled valine and proline. Peptide concentration was determined by amino acid analysis.

Tissues were stored frozen at −80°C in 15 ml Falcon tubes. Whole kidney tissues were submerged in liquid nitrogen for 5 minutes and kidney tissue was processed in a biopulverizer (MidSci, St. Louis, MO) to a fine powder. Pulverized tissue was transferred to a 5-ml glass dounce with a chilled spatula. Approximately 200 mg of tissue was used for peptide extraction. Four volumes of ice cold methanol (Burdick and Jackson, LC-MS Grade) were added to the tissue followed by a volume of water (Burdick and Jackson, LC-MS Grade) containing O-phenanthroline (Sigma Aldrich) and SIS peptides resulting in a final concentration of 1 mM and 1.8 nM, respectively. Tissue was homogenized in the dounce on ice for 1 minute. An aliquot of tissue homogenate was transferred to a 2-ml lo-bind microcentrifuge tube and stored at −20°C for at least 1 hour. Samples were further centrifuged for 10 minutes at 10,000×g at 4°C. An aliquot equal to approximately 60% of the total sample volume was transferred to a lo-bind tube equivalent to 100 mg of tissue (wet weight). From this aliquot, a volume equal to the wet weight of the starting tissue was transferred to a new lo-bind tube and diluted with ice cold water so that the final concentration of methanol was 40%. The total volume of this microcentrifuge tube was then transferred to a Microcon Ultracel YM-10 centrifugal filter (Milipore Ireland Ltd, Cork, IRL) following methods described by Lortie *et al.* [28]. Filters were preconditioned for 30 minutes with 40% methanol prior to use. Samples were filtered at 14,000×g for 1 hour at 4°C. The filtrate was transferred to a new lo-bind microcentrifuge tube. The filtrate volume was estimated by variable volume pipet and recorded to determine the fraction of starting volume collected. Two volumes of 0.1% formic acid were added to each sample. A solid phase extraction cartridge (Phenomenex Strata-x reversed phase, 60 mg, Torrance, CA) was conditioned with 1 ml acetonitrile and washed with 1 ml 0.1% formic acid. Peptides were bound to the column and washed with 1 ml 0.1% formic acid. Peptides were eluted sequentially in 12% Mobile Phase B [MPB, 95% acetonitrile (v/v), 0.2% formic acid (v/v)]/88% Mobile Phase A [MPA, 0.2% formic acid] and then 30% MPB/70% MPA to separate Ang-(1-7) from Ang-II [Ang-(1-8)], Ang-(2-10), and Ang-I [Ang-(1-10)]. Eluted peptides were flash frozen in liquid nitrogen, placed in a speedvac concentrator and dried. Peptides from the 12% MPB fraction were resuspended in MPA to a volume equivalent to 0.18 mg of starting kidney weight per microliter (approximately 100–150 microliters). Peptides from the 30% MPB fraction were resuspended in 10% MPB to the same volume equivalent. For each fraction, 10 µl of resuspended peptides were injected onto a 100 µm×2 cm C18 (100 Å with 5-µm particles) trap column (Acclaim PepMap 100; Thermo Fisher Scientific), and separated on a 75 µm×15 cm C18 (300 Å with 3 µm particles) analytical column (Acclaim PepMap 100; Thermo Fisher Scientific). Reverse phase separation was performed with a gradient of 0% to 40% MPB over 42 minutes on a 2D+ NanoLC system (Eksigent, Dublin, CA). The nanoLC was coupled to a TripleTOF 5600 mass spectrometer (AB Sciex, Foster City, CA) through a nanospray source. Declustering potential was set at 80 V. For parallel reaction monitoring (PRM) quantification, the dominant charge state of each peptide was determined and collision energies were optimized manually for SIS peptides (Table 1). Peptide data was collected in positive ion mode using product ion scans (set at 1 unit resolution). External calibration curves were constructed for each SIS peptide. Seven concentrations were injected from 0.3 fmols to 25 fmols in duplicate, with the exception of Ang-II where 20 fmols was injected as the highest concentration. Measurements were calculated by using area under the curve for ion pairs using the MQ4 algorithm built in to MultiQuant (v 2.0.2, AB Sciex). Fragment ions were extracted from each product scanusing a monoisotopic mass ±0.1 m/z. Regression was performed using peak areas with linear fit and 1/x weighting. Limit of detection and lower limit of quantification (LLOQ) were calculated for SIS peptides as 3 and 10 standard deviations of the mean from triplicate kidney lysate preps without SIS peptides. Percent coefficient of variation was calculated for a single sample injected in triplicate.

**Table 1:** LC/MS/MS peptide transition ions, optimized collision energy and standard curve correlation coefficients.

Peptide	Peptide Sequence	Charge state	Product scan (m/z)	Extracted Ion Monitored	Collision Energy (V)	*R*	LOD (fmol)	LLOQ (fmol)	CV%
Ang-I	DRVYIHPFHL	(M+3H)+3	432.9	b5, 647.35	25				14.4
Ang-I∧	DRV∧YIHP∧FHL	(M+3H)+3	438.2	b5, 657.35	25	0.995	0.012	0.030	
Ang-(2-10)	RVYIHPFHL	(M+3H)+3	394.6	b4, 532.32	23				2.1
Ang-(2-10)∧	RV∧YIHPFHL	(M+3H)+3	396.6	b4, 538.32	23	0.991	1.367	3.809	
Ang-II	DRVYIHPF	(M+3H)+3	349.5	b3, 371.20	14				5.9
Ang-II∧	DRV∧YIHPF	(M+3H)+3	351.5	b3, 377.20	14	0.993	0.005	0.015	
Ang-(1-7)	DRVYIHP	(M+3H)+3	300.5	b3, 371.20	14				18.4
Ang-(1-7)∧	DRVYIHP	(M+3H)+3	302.5	b3, 377.20	14	0.986	0.017	0.046	

On column limit of detection (LOD), On column lower limit of quantification (LLOQ) and concentration range measured for peptides in this study. ∧ indicates ^13^C/^15^N SIS peptide. CV = coefficient of variation.

### Statistical analyses

Blood pressure data were analyzed by repeated measures ANOVA. Differences within groups were analyzed using paired t-tests adjusted with Bonferroni modification. Differences between group means at each time point were tested using one-way ANOVA followed by Tukey-Kramer *post-hoc* test. Urinary protein excretions were log10 transformed and tested using a two-way ANOVA for repeated measures followed by Scheffe *post hoc* test for differences between the groups. Untransformed 24-hour urinary protein excretions at each time point were then tested using Kruskal-Wallis test. If a significant main effect was detected then differences between groups were tested using the Mann-Whitney U test with the probability adjusted using the Bonferroni modification. Urine sodium was also assessed by two-way ANOVA for repeated measures and pairwise multiple comparisons were made using the Holm-Sidak method. Multiple comparisons of Ang peptide levels were made using ANOVA when data were normally distributed and with log10 transformation when non-normally distributed. *Post-hoc* pairwise comparisons were made using the Holm-Sidak method. Correlations were assessed using Spearman Rank Order correlation. For Ang peptide measurements by LC/MS/MS, only values above the LLOQ were utilized for statistical comparisons.

## Results

### Ang Peptide Binding

**Figure 2:**
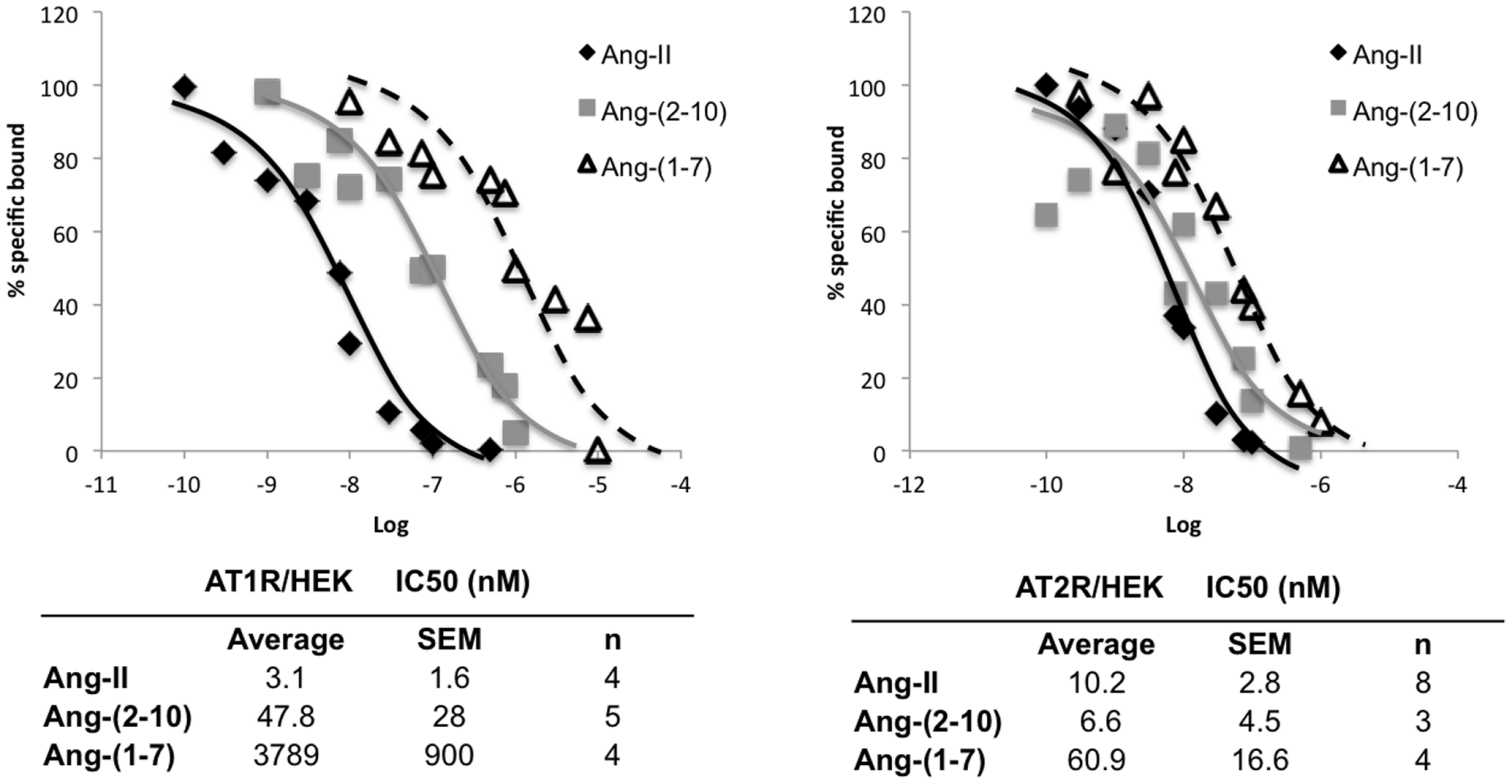
Displacement curves of competition radioligand binding studies. Representative curves are shown for each peptide from studies performed on cells expressing the indicated Ang receptor. Average IC50 values ± SEM from the indicated number of studies (n) are shown.

As illustrated in Figure 2, all three ligands demonstrated concentration-dependent displacement of radiolabeled Ang-II from both AT_1_ and AT_2_ receptors indicating specific interaction with the receptors in HEK-293 cells. However, the rank order potency for the ligands differed between the two expressed receptors. For the AT_1_ receptor, the rank order for affinity was: Ang-II>Ang-(2-10)>>Ang-(1-7). On the other hand, for the AT_2_ receptor, Ang-II and Ang-(2-10) demonstrated similar affinity for the receptor, which was approximately 6–10 greater than that of Ang-(1-7). In addition, Ang-(1-7) appeared to demonstrate a greater affinity for the AT_2_ receptor than for the AT_1_ receptor, as reflected by an IC50 value approximately 60-fold higher for the AT_1_ receptor.

### Effect of Ang-(1-7) and Ang-(2-10) on a rat model of FSGS

**Figure 3:**
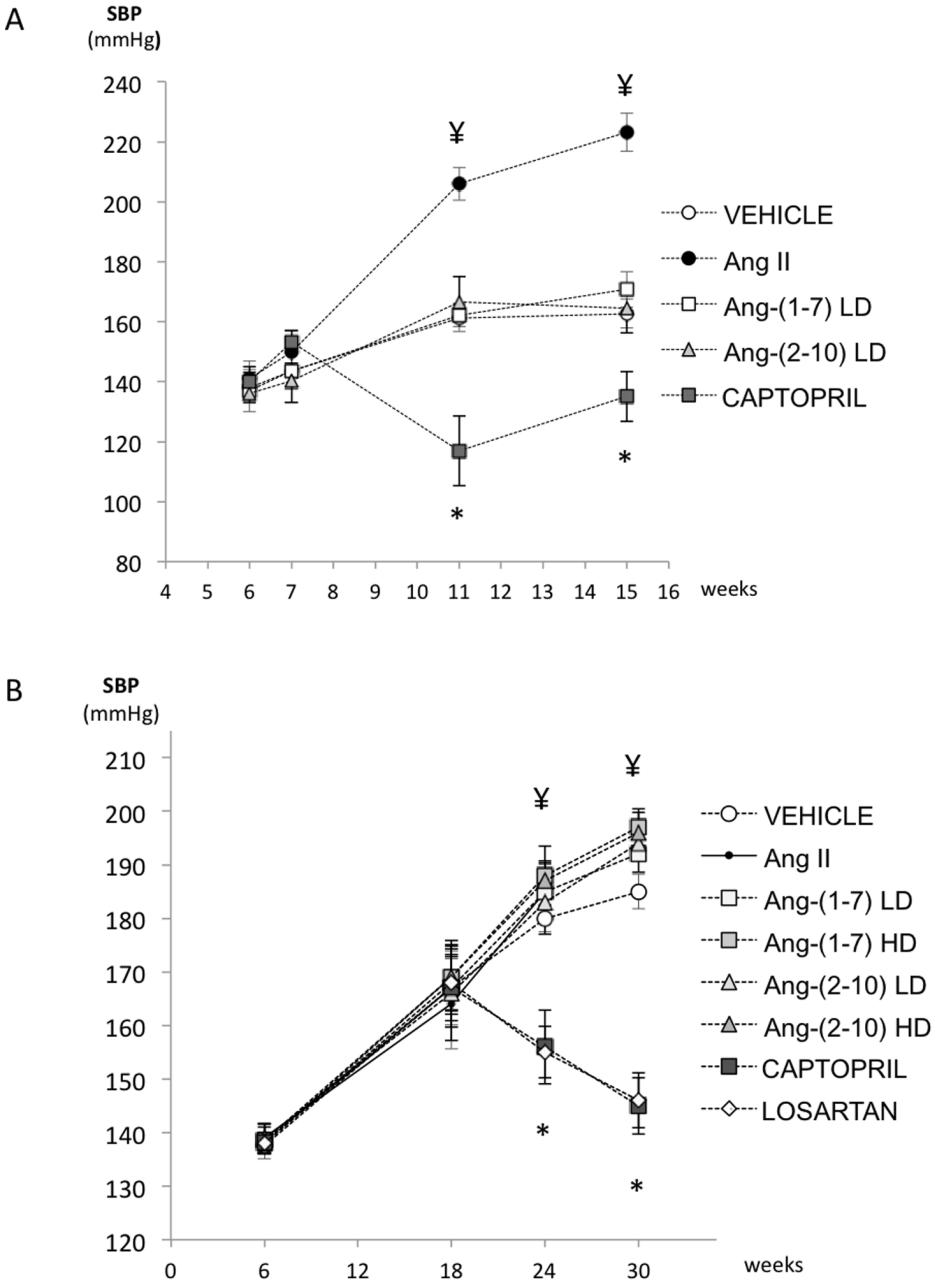
Effect of Ang peptides on blood pressure. Tail-cuff systolic blood pressure readings in uni-nephrectomized FHH rats obtained in *A*: *early disease protocol [n = 4/group, except captopril group (n = 5)]:* at the time of uni-nephrectomy (week 6), initial mini-pump implantation (week 7), mini-pump exchange (week 11), and sacrifice (week 15); or *B*: *late disease protocol [n = 9/group, except vehicle group (n = 7)]:* at the time of uni-nephrectomy (week 6), initial mini-pump implantation (week 18), mini-pump exchange (week 24), and sacrifice (week 30). * = significantly lower than vehicle (p = 0.014); ¥ = significantly higher than vehicle (p<0.001). Only 2 rats from the Ang-(2-10) HD group and none of the Ang-II group were alive at week 30. Data are in means ± SEM. SBP = systolic blood pressure, LD = low-dose, HD = high-dose.

In accordance to previous studies utilizing the uni-nephrectomized FHH rat model [25], [29], all animals exhibited a progressive increase over time in systemic blood pressure, urinary protein excretion and parenchymal injury characterized by focal segmental glomerulosclerosis and microcystic tubular dilatation. In the late disease model, approximately 80% of the glomeruli were affected with some degree of glomerulosclerosis, either segmental or global, demonstrating the robustness of the animal model. The changes observed in systolic blood pressure during the early and late disease models are shown in Figure 3. Data on changes in body weight, kidney weight and urine volume are presented in Table 2.

**Table 2:** Body weight, kidney weight and urine volumes at initiation of therapy and study end.

	Body Wt (g)		Kidney Wt (g)	Kid. Wt/B. Wt (g/100 g b.wt)	Urine Volume (ml/day)	
*Early Disease Model [n = 4/group, except captopril group (n = 5)]*						
	Baseline (week 7)	Final (week 15)	Final (week 15)		Baseline (week 7)	Final (week 15)
Vehicle (0.9% NaCl)	175 (13)	320 (18)	1.9 (0.3)	0.6 (0.0)	31.4 (6.2)	28.4 (2.7)
Ang-II	185 (43)	323 (18)	2.0 (0.2)	0.7 (0.1)	39.7 (15.1)	37.6 (14.1)
Ang-(1-7) LD	176 (23)	317 (22)	2.0 (0.2)	0.6 (0.1)	34.8 (10.4)	28.5 (2.3)
Ang-(2-10) LD	160 (17)	299 (23)	1.9 (0.2)	0.7 (0.1)	30.9 (6.3)	30.9 (5.3)
Captopril	158 (3)	284 (10)	1.6 (0.1)	0.5 (0.0)	30.9 (2.3)	31.5 (1.0)

*Late Disease Model [n = 9/group, except vehicle (n = 7) and final Ang-(2-10) HD group (n = 2)]*						
	Baseline (week 18)	Final (week 30)	Final (week 30)		Baseline (week 18)	
Vehicle (0.9% NaCl)	340 (16)	340 (43)	2.0 (0.4)	0.6 (0.1)	27.9 (5.4)	37.6 (6.4)
Ang-(1-7) LD	342 (13)	344 (21)	2.1 (0.2)	0.6 (0.1)	32.6 (7.6)	36.7 (11.4)
Ang-(1-7) HD	341 (23)	340 (22)	2.0 (0.3)	0.6 (0.0)	31.5 (5.6)	40.4 (9.7)
Ang-(2-10) LD	340 (33)	336 (41)	2.1 (0.2)	0.6 (0.1)	32.1 (6.7)	43.5 (5.5)
Ang-(2-10) HD	350 (22)	339 (8)	1.6 (0.6)	0.5 (0.4)	30.7 (6.6)	40.2 (7.8)
Captopril	338 (15)	354 (16)	1.9 (0.1)	0.5 (0.1)	28.5 (5.3)	31.6 (11)
Losartan	339 (14)	351 (22)	2.1 (0.1)	0.6 (0.1)	29.0 (4.8)	35.1 (8)

Data are in means (SD). Wt: weight, B: body, Kid: kidney, LD: low-dose, HD: high-dose.

**Figure 4:**
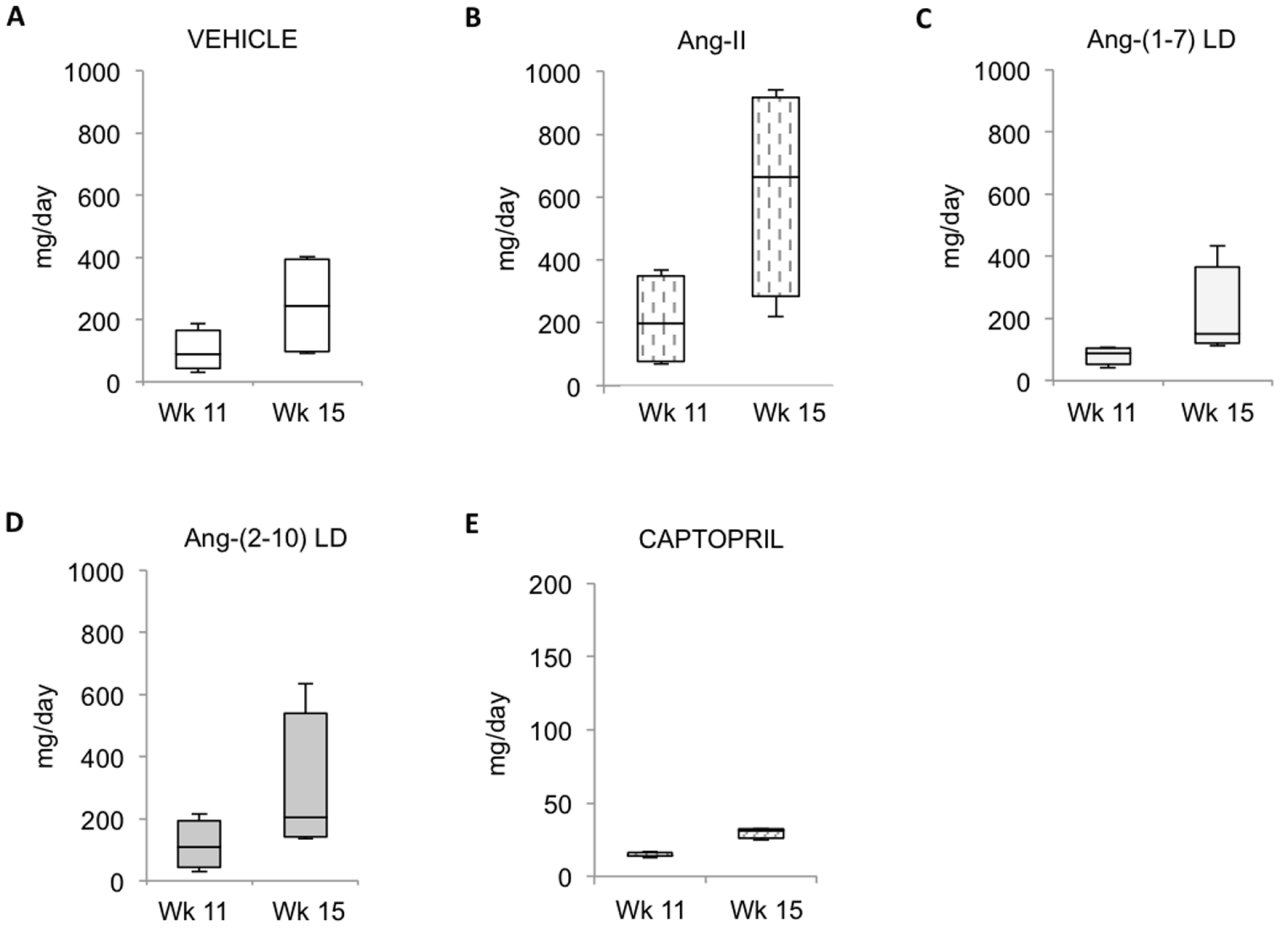
Effect of Ang peptides on proteinuria during the early disease protocol. Urinary protein excretion measured in uni-nephrectomized FHH rats treated with: *A*: vehicle; *B*: Ang-II; *C*: low-dose Ang-(1-7); *D*: low-dose Ang-(2-10); and *E*: captopril; for 8 weeks, i.e., between weeks 7 and 15 after birth; n = 4/group, except captopril group (n = 5). LD = low-dose, HD = high-dose.

**Figure 5:**
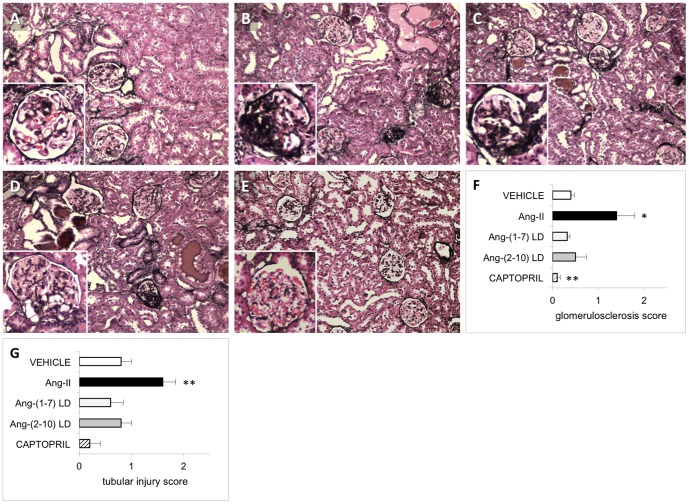
Effect of Ang peptides on renal histology during the early disease protocol. Representative light microscopy (10×) images of kidney cortex specimens of uni-nephrectomized FHH rats double-stained with hematoxylin-eosin (H&E) and Jones silver stain. Tissue was harvested at the end of the early disease protocol, i.e., after 8 weeks of treatment with: *A*: vehicle; *B*: Ang II; *C*: Ang-(1-7) LD; *D*: Ang-(2-10) LD; and *E*: captopril. *F*: Glomerulosclerosis scores. *G*: Tubular injury scores. Insets show a representative glomerulus. Segmental glomerulosclerosis and microcystic tubular dilatation is appreciated in all treatment groups. * = p<0.01 compared to vehicle; ** = p<0.05 compared to vehicle n = 4/group, except captopril group (n = 5). Data are in means ± SEM. LD = low-dose.

In the early disease model, rats in all groups exhibited a small but consistent elevation in systolic blood pressure one week after single nephrectomy. Four weeks after initiation of therapy, systolic blood pressure in vehicle-treated rats increased (from 144±5, 161±4 mmHg, p = 0.01) and it remained elevated at 8 weeks (162±4 mmHg). A similar rise in arterial blood pressure was observed among animals treated with either low-dose Ang-(1-7) or low-dose Ang-(2-10). Ang-II induced a broader rise in mean systolic blood pressure compared to that in vehicle-treated rats after both 4 and 8 weeks of treatment. As expected, captopril decreased systolic blood pressure after 4 and 8 weeks of therapy (117±5 and 134±3, respectively); (p<0.0001 vs. vehicle) (Fig. 3A). Urine sodium excretion did not change over time in any of the groups, nor was a difference found between groups (Table S1). Urinary protein excretion increased between 4 and 8 weeks after the commencement of treatment (p<0.001). Median proteinuria in vehicle-treated rats was 89 (31–188) and 243 (91–402), mg/day, after 4 and 8 weeks, respectively (Fig. 4). Captopril significantly mitigated the development of proteinuria [14 (13–17) and 30 (25–33) mg/day, after 4 and 8 weeks of therapy, respectively; p = 0.014 vs. vehicle]. In contrast, proteinuria was not modified by either Ang-(1-7) or Ang-(2-10) at the doses tested and it was increased by Ang-II. Ang-II significantly worsened the degree of glomerulosclerosis (1.4±0.4 vs. 0.4±0.1; p<0.01) and tubular injury (1.6±0.2 vs. 0.8±0.2; p<0.05) compared to vehicle, whereas neither Ang-(1-7) nor Ang-(2-10) infusion changed the degree of structural damage. Captopril caused a significant reduction in glomerulosclerosis (0.1±0.1 vs. 0.4±0.1; p<0.05) but a non-significant reduction in tubular injury (Fig. 5).

**Figure 6:**
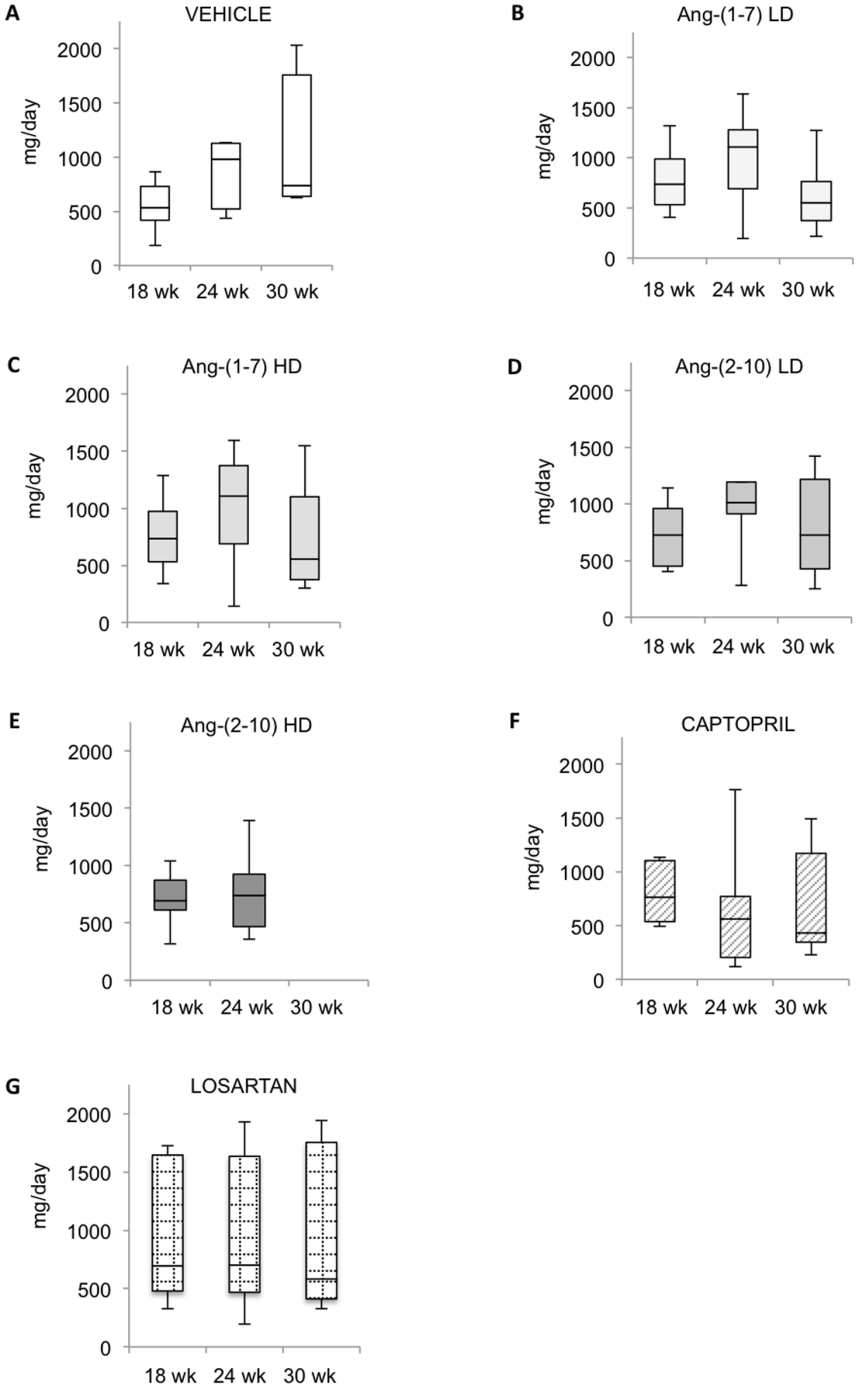
Effect of Ang peptides on proteinuria during the late disease protocol. Urinary protein excretion measured in uni-nephrectomized Fawn-Hooded Hypertensive rats treated with: *A:* vehicle; *B:* low-dose Ang-(1-7); *C:* high-dose Ang-(1-7); *D:* low-dose Ang-(2-10); *E:* high-dose Ang-(2-10); *F:* captopril and *G:* losartan for 12 weeks, i.e., between weeks 18 and 30 after birth*;* n = 9/group, except vehicle group (n = 7). LD = low-dose, HD = high-dose.

**Figure 7:**
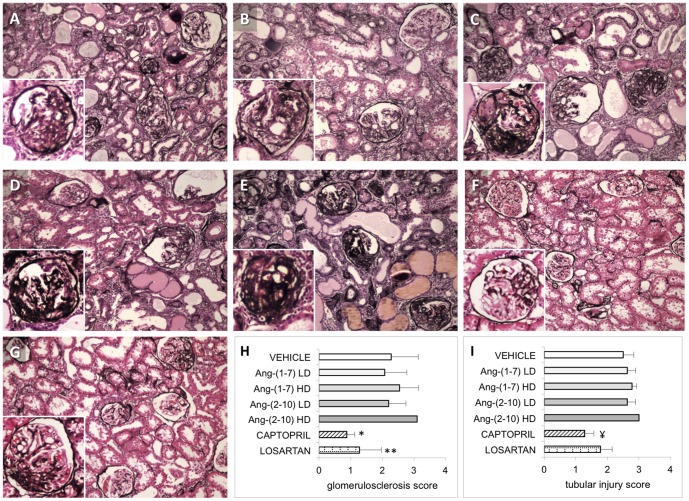
Effect of Ang peptides on renal histology during the late disease protocol. Representative light microscopy (10×) images of kidney cortex sections of uni-nephrectomized Fawn-Hooded Hypertensive rats double-stained with hematoxylin-eosin (H&E) and Jones silver stain. Tissue was harvested at week 30, i.e., after 12 weeks of treatment with: *A:* vehicle; *B:* Ang-(1-7) LD; *C:* Ang-(1-7) HD; *D:* Ang-(2-10) LD; *E:* Ang-(2-10) HD; *F:* captopril; *G:* losartan; *H:* glomerulosclerosis scores; *I:* tubular injury scores. Insets show a representative glomerulus. Significant segmental and global glomerulosclerosis, glomerular collapse and microcystic tubular dilatation is appreciated in all treatment groups, except in captopril-treated and losartan-treated rats. * = p<0.0001 compared to vehicle; ** = p<0.005 compared to vehicle; ¥ = p<0.05 compared to vehicle; n = 9/group except for vehicle group (n = 7) and Ang-(2-10) HD (n = 2). Data are in means ± SEM. LD = low-dose, HD = high-dose.

In the late disease model, all animals exhibited a substantial increase in systolic blood pressure 12 weeks after single nephrectomy (Fig. 3B). Thereafter, vehicle-treated rats continued to increase their blood pressure, raising it from 167±8 (at pump implantation) to 179±2 mmHg (after 6 weeks) (p<0.005) and maintaining it elevated at 185±3 mmHg after 12 weeks. Captopril and losartan decreased systolic blood pressure (156±7 and 155±5 mmHg at 6 weeks, and 145±5 and 146±5 mmHg after 12 weeks; for captopril and losartan, respectively; p<0.0001 vs. vehicle), suggesting that hypertension in FHH rats is partially Ang-II mediated. In contrast, low-dose Ang-(1-7) had no effect on blood pressure, low-dose Ang-(2-10) raised blood pressure after 12 weeks (194±3 mmHg; p<0.01 vs. vehicle), and both high-dose Ang-(1-7) and high-dose Ang-(2-10) raised blood pressure throughout the study (188±3 and 187±3 mmHg after 6 weeks, and 197±3 and 196±4 mmHg; for high-dose Ang-(1-7) and high-dose Ang-(2-10), respectively; p<0.001 vs. vehicle). However, only 2 high-dose Ang-(2-10)-treated rats survived until the end of the study. Ang-II-treated rats also exhibited increased blood pressure after 6 weeks, but no rats in this group survived beyond that time mark. Although the urine sodium excretion decreased in all groups after the 12-week therapy, no differences were found between groups (Table S1). Median proteinuria in vehicle-treated rats was 2 (1–5), 537 (184–862), 978 (433–2839) and 738 (622–2032) mg/day, at baseline (week 6), at the time of pump implantation (week 18), at week 24 and at week 30, respectively (Fig. 6). There was no change in 24-hour urinary protein excretion over the 12 weeks of treatment for any of the groups. However, the progression of the degree of proteinuria between weeks 18 and 24 was significantly attenuated by captopril (+342±289 vs. −225±453 mg/day, vehicle vs. captopril, respectively; p = 0.012). Proteinuria was not modified by either Ang-(1-7) or Ang-(2-10), regardless of dosing. Captopril significantly reduced the degree of glomerulosclerosis (0.88±0.2 vs. 2.29±0.9, p<0.0001) and tubular injury score (2.6±0.4 vs. 1.3±0.4, p<0.05) compared to vehicle, whereas losartan only reduced glomerulosclerosis (1.28±0.7 vs. 2.29±0.9, p<0.0005). None of the Ang-(1-7) and Ang-(2-10) treatments had an effect on structural damage (Fig. 7).

### Ang Peptide Content

**Figure 8:**
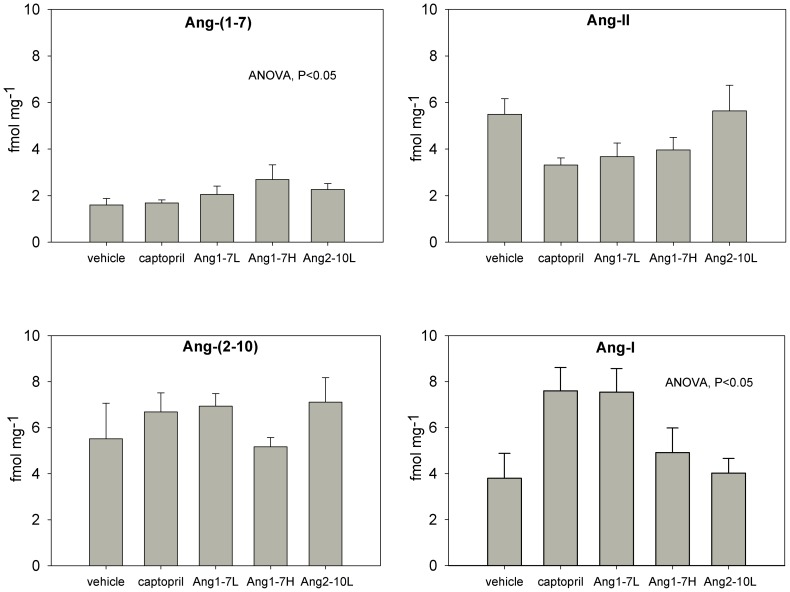
Intrarenal Ang peptide content as measured by Parallel Reaction Monitoring. Kidneys were harvested at the end of the late disease protocol (after 12 weeks of therapy). Treatment arms are listed in the *x*-axes. ANOVA (p<0.05) indicates differences between groups that were detected, but no pairwise differences were significant; n = 5/group Data are in means ± SEM. L = low-dose, H = high-dose.

The concentration of Ang peptides in kidney tissue was measured in kidneys harvested upon completion of the late disease model protocol, i.e., at 30 weeks of age, after 12 weeks of therapy. LC/MS/MS assay performance measures and peptide concentrations are summarized in Table 1 and Figure 8. Kidney Ang-I and Ang-(1-7) concentrations were different between groups, but evaluation by *post-hoc* comparison did not reveal any significant differences. Kidney levels of Ang-II and Ang-(2-10) were not different between groups. The statistical power of the test for both Ang-II and Ang-(2-10) were 0.27 and 0.05, respectively. When Ang-II levels were compared separately between vehicle and captopril groups using a Mann-Whitney U test, they were found to be lower in captopril-treated rats (p = 0.002) suggesting that the multi-group comparisons were underpowered. Because the majority of rats treated with high-dose Ang-(2-10) did not survive, we were unable to determine the effect of high-dose Ang-(2-10) treatment on Ang-(2-10) kidney content. Table S2 shows the range of previously reported intrarenal Ang peptide concentration in comparison to this study [17], [30]–[34].

**Figure 9:**
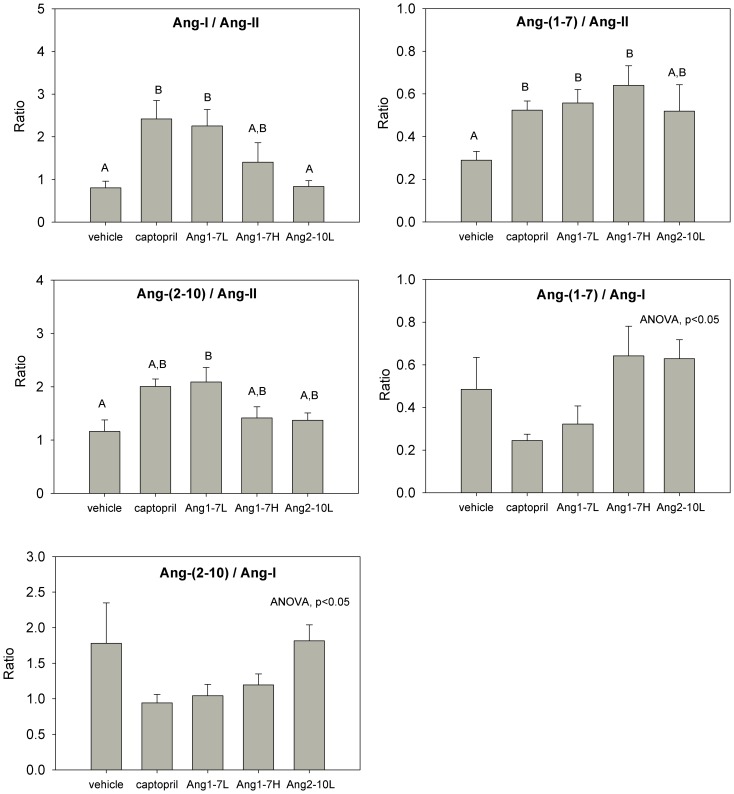
Ratios of intrarenal Ang peptide abundance. Kidneys were harvested at the end of the late disease protocol (after 12 weeks of therapy). Treatment arms are listed in the *x*-axes. Group specific *post-hoc* pairwise differences (P<0.05) are indicated by letters. Equivalent letters indicate groups that are not significantly different from each other and different letters indicate a significant difference was detected between groups. ANOVA (p<0.05) indicates if differences between groups were detected, but no pairwise differences were significant; n = 5/group Data are in means ± SEM. L = low-dose, H = high-dose.

Although individual Ang-(1-7) peptide levels were not elevated in the Ang-(1-7) infused groups, the ratio of Ang-(1-7)/Ang-II content was higher in both the low dose and high dose groups compared to vehicle (Figure 9), suggesting an effect of Ang-(1-7) infusion on Ang peptide balance in the kidney, but were not different from captopril treatment, a finding that is in line with a known effect of ACE inhibition on Ang-(1-7) degradation and Ang-II generation [32]. Low-dose Ang-(1-7) infusion also resulted in an elevation in the Ang-I/Ang-II and Ang-(2-10)/Ang-II ratios compared to vehicle-infused controls (p = 0.01) but was not different from captopril-treated animals. The effects of Ang-(1-7) and captopril on Ang-(1-7)/Ang-I and Ang-(2-10)/Ang-I ratios could not be discerned by *post hoc* comparison, despite the fact that significant differences between groups were detected (ANOVA, p<0.05). Infusion of low-dose Ang-(2-10) did not significantly change any ratio compared to vehicle.

In agreement with the pathogenesis of FSGS, we found a significant correlation between proteinuria and increase in kidney size, likely from glomerulomegaly (Table S3). We also found a significant correlation between systolic blood pressure and urinary volume, possibly reflecting pressure natriuresis. In terms of Ang peptide content, higher Ang-II abundance was associated with increased kidney size and urinary volume, whereas Ang-(1-7) exhibited an even stronger correlation with urinary volume, possibly due to its reported aquaretic property [35]. No Ang peptide level correlated with the degree of proteinuria.

## Discussion

Our study demonstrates that chronic intravenous administration of Ang-(1-7) or Ang-(2-10) does not ameliorate glomerulosclerosis in a spontaneous rat model of FSGS. The lack of benefit of these alternative Ang peptides was observed regardless of the delivered dose or the stage of the disease studied. We opted to infuse the peptides longer that previously done by others in order to allow more time for the peptide to elicit a measurable effect. However, no reduction in glomerular injury was observed after 8 weeks of infusion during the early disease protocol or after 12 weeks of infusion during the late disease protocol, for either Ang-(1-7) or Ang-(2-10). Our study is the first to evaluate the long-term effect of 2 doses of Ang-(1-7) in a model of spontaneous FSGS.

Significant enthusiasm was raised by a body of evidence suggesting potentially beneficial cardiovascular effects of Ang-(1-7) in various *in vitro* and *in vivo* models of heart and kidney disease [36], [37]. Those studies demonstrated that Ang-(1-7) can elicit biological actions that are antagonistic to those of Ang-II. Stimulated by those observations, many laboratories have searched for a renoprotective effect of Ang-(1-7) in various rodent models of human glomerular disease [18]. The majority of those studies administered Ang-(1-7) via osmotic mini-pump at a dose of 400 ng/kg/min, whereas others used 100 ng/kg/min, doses that we categorized as high and low, respectively. Overall, the results obtained across all studies have not been uniform. In a study where Ang-(1-7) was delivered via a route similar to that employed in this study, i.e., intravenously, for 2 weeks, Ang-(1-7) had no effect on either blood pressure or proteinuria in a rat model of adriamycin-induced nephrosis [16]. In a mouse remnant kidney model of progressive glomerulopathy, mesangial expansion was worsened after 4 weeks of subcutaneous administration of Ang-(1-7), while blood pressure remained unaffected [4]. In a rat remnant kidney model, blood pressure was increased by Ang-(1-7) delivered subcutaneously [38]. Proteinuria was not altered in either of the remnant kidney studies. In contrast, in a model of mesangioproliferative glomerulonephritis, anti-Thy1.1 nephritis, Ang-(1-7) ameliorated the degree of glomerular scarring and decreased proteinuria [14]. Furthermore, the reported effects of Ang-(1-7) infusion on models of diabetic kidney disease have been conflicting. Benter *et al.* reported a decrease in blood pressure and proteinuria after 4 weeks of intraperitoneal Ang-(1-7) in spontaneously hypertensive rats injected with streptozotocin [39], whereas Shao *et al.* observed worsening proteinuria and transforming growth factor beta accumulation after 6 weeks of intravenous Ang-(1-7) in streptozotocin-induced diabetic rats [17]. Differences in methodology may account for some of the discrepancy among studies, including dose of the peptide, route of administration, duration of therapy and disease model. However, it remains difficult to reconcile the findings across laboratories. Furthermore, Ang-(1-7) has been reported to both attenuate [40] and promote [41] profibrotic pathways though *mas* receptor stimulation in cultured mesangial cells. Therefore, glomerular cells may exhibit variable responses to Ang-(1-7) depending on the experimental conditions.

Despite the vasodilatory effect of Ang-(1-7) reported by others [42], [43], we did not observe a reduction in systolic blood pressure in Ang-(1-7)-infused animals. On the contrary, animals treated with high-dose Ang-(1-7) experienced an increase in blood pressure. Previous studies also found pressor effect of Ang-(1-7) in rat models of kidney disease [38], [44]. Our radioligand binding curves indicate that Ang-(1-7) has low affinity for the AT_1_ receptor. However, it is conceivable that at the administered pharmacological doses, Ang-(1-7) may have elicited some degree of AT_1_ receptor stimulation, thereby explaining the observed rise in systolic blood pressure. However, such mechanism remains speculative. Alternatively, binding affinity might change in disease states. Others have reported that Ang-(1-7) is capable of binding the AT_1_ receptor [45]. We did not treat animals simultaneously with Ang-(1-7) and losartan to determine if the pressor effect of Ang-(1-7) could be reduced by AT_1_ receptor blockade. However, even under those circumstances, it would be difficult to ascertain whether the observed pressor effect of Ang-(1-7) is indeed AT_1_ receptor-mediated because losartan alone reduced blood pressure in our animals. Because a *mas* receptor antagonist was not utilized, it cannot be determined whether the observed pressor effect of Ang-(1-7) was *mas* receptor-mediated.

Stimulation of the AT_2_ receptor may mitigate parenchymal injury in models of renal disease by antagonizing the pro-inflammatory effects of Ang-II [46]. Thus, the observed higher affinity of Ang-(2-10) for the AT_2_ receptor compared to the AT_1_ receptor led us to postulate that preponderance of AT_2_ stimulation may result in a renoprotective effect in our rat model of FSGS. However, there was no significant effect of Ang-(2-10) infusion at a dose of 100 ng/kg/min, neither early nor late in the disease, on blood pressure, proteinuria or histology. Furthermore, when given at a higher dose of 400 ng/kg/min, Ang-(2-10) raised systolic blood pressure and led to the demise of 7 out of the 9 treated animals. Therefore, we speculate that at the higher administered pharmacological doses, Ang-(2-10) could potentially stimulate AT_1_ receptors. However, further studies will be necessary to verify this hypothesis.

Because this investigation aimed to induce a local effect in the kidneys by infusion of Ang-(1-7) or Ang-(2-10), we quantified the kidney content of a panel of Ang peptides to determine whether the intravenous delivery of the Ang peptides led to a corresponding increase in their concentration in the kidney. We utilized PRM (nano-flow LC-MS/MS) as an analytical technique for peptide quantification because of its superior specificity compared to antibody-based methods. Although there appear to be a dose-dependent increase in Ang-(1-7) kidney content in Ang-(1-7)-treated rats, no significant difference was found when the content of the heptapeptide was compared to that of vehicle-treated rats. Therefore, the intravenous route may be an inadequate strategy to elevate Ang peptide content in the kidney. However, because whole kidney homogenates were analyzed, our measurements may have underestimated actual changes in cortical Ang peptide concentrations. In addition, plasma Ang peptide concentration was not measured to verify whether pharmacological concentrations of the delivered peptides were reached. Furthermore, it is not known whether the local concentration of Ang peptides is altered in FHH rat kidneys as the kidney disease evolves. Therefore, intrinsic abundance of endogenous Ang peptides in injured FHH rat kidneys may comprise a large fraction of Ang peptide concentration relative to the delivered amount of Ang-(1-7) or Ang-(2-10).

In summary, chronic intravenous administration of Ang-(1-7) or Ang-(2-10) failed to ameliorate glomerular damage in a rat model of FSGS. The lack of benefit might be related to an undesired hypertensive effect of the peptides and/or suboptimal peptide delivery to the kidney attained with intravenous delivery.

## Supporting Information

Table S1 **Urine sodium excretion rates.**
(DOCX)

Table S2 **Intrarenal Ang peptide concentrations gathered from previous publications for comparison with this study.** Data from Nishiyama et al. were extracted from supplementary table and from the text of the results section and included all pharmacological manipulations. Range of means ± SE were extracted when possible. RIA, Radioimmunoassay; LC/MS/MS, liquid chromatography tandem mass spectrometry; HPLC, High Performance Liquid Chromatography. * Seikaly et al. reported immunoreactive angiotensin peptides of which 23% was considered to be Ang-II.
(DOCX)

Table S3 **Spearman Rank correlations between Ang peptide content and phenotypical parameters.** Significant correlations are listed for p<0.05.
(DOCX)
