# Supplementary material for: Lack of Renoprotective Effect of Chronic Intravenous Angiotensin-(1-7) or Angiotensin-(2-10) in a Rat Model of Focal Segmental Glomerulosclerosis
Source: PLoS One. 2014 Oct 22;9(10):e110083. doi: 10.1371/journal.pone.0110083 (PMC4206519; doi:10.1371/journal.pone.0110083)
Supplement: Table S1 — Urine sodium excretion rates. (DOCX) [file pone.0110083.s001.docx]

**Table S1: Urine sodium excretion rates.**

|  | **Urine sodium (mg/day)** | |
| --- | --- | --- |
| ***Early disease model [n=4/group, except captopril group (n=5)]*** | | |
|  | **Baseline (week 7)** | **Final (week 15)** |
| Vehicle (0.9% NaCl) | 47.5 (12) | 33.6 (14) |
| Ang-II | 47.6 (14) | 42.6 (6) |
| Ang-(1-7) LD | 31.6 (11) | 37.3 (11) |
| Ang-(2-10) LD | 35.9 (15) | 37.4 (6) |
| Captopril | 43.9 6) | 36.3 (13) |
| ***Late disease model [n=9/group, except vehicle (n=7) and final Ang-(2-10) HD (n=2)]*** | | |
|  | **Baseline (week 18)** | **Final (week 30)** |
| Vehicle (0.9% NaCl) | 55.1 (9) | 37.3 (12) |
| Ang-(1-7) LD | 69.1 (20) | 31.9 (17) |
| Ang-(1-7) HD | 64.9 (15) | 40.1 (15) |
| Ang-(2-10) LD | 63.5 (14) | 29.8 (13) |
| Ang-(2-10) HD | 75.1 (9) | 36.9 (3) |
| Captopril | 60.3 (60) | 36.1 (12) |
| Losartan | 62.7 (27) | 36.5 (13) |
