# Supplementary material for: Lack of Renoprotective Effect of Chronic Intravenous Angiotensin-(1-7) or Angiotensin-(2-10) in a Rat Model of Focal Segmental Glomerulosclerosis
Source: PLoS One. 2014 Oct 22;9(10):e110083. doi: 10.1371/journal.pone.0110083 (PMC4206519; doi:10.1371/journal.pone.0110083)
Supplement: Table S2 — Intrarenal Ang peptide concentrations gathered from previous publications for comparison with this study. Data from Nishiyama et al. were extracted from supplementary table and from the text of the results section and included all pharmacological manipulations. Range of means ± SE were extracted when possible. RIA, Radioimmunoassay; LC/MS/MS, liquid chromatography tandem mass spectrometry; HPLC, High Performance Liquid Chromatography. * Seikaly et al. reported immunoreactive angiotensin peptides of which 23% was considered to be Ang-II. (DOCX) [file pone.0110083.s002.docx]

**Table S2: Intrarenal Ang peptide concentrations gathered from previous publications for comparison with this study.** Data from Nishiyama et al. were extracted from supplementary table and from the text of the results section and included all pharmacological manipulations. Range of means ± SE were extracted when possible. RIA, Radioimmunoassay; LC/MS/MS, liquid chromatography tandem mass spectrometry; HPLC, High Performance Liquid Chromatography. * Seikaly et al. reported immunoreactive angiotensin peptides of which 23% was considered to be Ang-II.

|  |  |  |  | **peptide concentration (fmol/mg)** | | | |
| --- | --- | --- | --- | --- | --- | --- | --- |
| **Study** | **Rat strain** | **Sample Type** | **Assay** | **Ang-I** | **Ang-(2-10)** | **Ang-II** | **Ang-(1-7)** |
| Seikaly et al. 1990 | Munich-Wistar | Glomerular Filtrate | HPLC/RIA | - | - | 32.8* | - |
| Seikaly et al. 1990 | Munich-Wistar | StarVessel Kidney | HPLC/RIA | - | - | 95.4* | - |
| Campbell et al. 1990 | Sprague-Dawley | Kidney Tissue | HPLC/RIA | 0.138 - 0.234 | 0.034 - 0.041 | 0.048 - 0.338 | 0.027 - 0.033 |
| Navar et al | Sprague-Dawley | Kidney Tissue | HPLC/RIA | 1.28 | - | 0.97 | - |
| Nishiyama et al. 2002 | Sprague-Dawley | Kidney Tissue | RIA | 0.178 | - | 0.19 | - |
| Nishiyama et al. 2002 | Sprague-Dawley | Interstitial Dialysis | RIA | 0.23 - 0.85 | - | 0.51 - 3.77 | - |
| Shao et al, 2008 | Sprague-Dawley | Kidney Tissue | RIA | - | - | 6.0 - 11.2 | - |
| Prieto et al. 2011 | Goldblatt | Kidney Tissue | HPLC | 0.02 - 0.08 | N/A | 0.1 -0.6 | 0.02 - 0.08 |
| This study | Fawn-hooded | Kidney Tissue | LC/MS/MS | 2.21 - 10.87 | 2.86 - 10.24 | 1.92 - 9.43 | 0.82 - 4.97 |
