# Supplementary material for: Lack of Renoprotective Effect of Chronic Intravenous Angiotensin-(1-7) or Angiotensin-(2-10) in a Rat Model of Focal Segmental Glomerulosclerosis
Source: PLoS One. 2014 Oct 22;9(10):e110083. doi: 10.1371/journal.pone.0110083 (PMC4206519; doi:10.1371/journal.pone.0110083)
Supplement: Table S3 — Spearman Rank correlations between Ang peptide content and phenotypical parameters. Significant correlations are listed for p<0.05. (DOCX) [file pone.0110083.s003.docx]

**Table S3: Spearman Rank correlations between Ang peptide content and phenotypical parameters.** Significant correlations are listed for p<0.05.

|  |  |  | Variable 1 |  |
| --- | --- | --- | --- | --- |
| Variable 2 |  | kidney wt ▪ body wt^-1^ | Uv | BP |
| Ang-(1-7) |  |  | 0.472 (p<0.01) | 0.347 (p<0.05) |
| Ang-I |  | 0.355 (p<0.05) |  |  |
| Ang-II |  | 0.394 (p<0.05) | 0.348 (p<0.05) |  |
| Ang-I/Ang-II |  |  | 0.404 (p<0.05) |  |
| Ang-I / Ang-(1-7) |  |  | 0.498 (p<0.01) | 0.354 (p<0.05) |
| Ang-(2-10) / Ang-II |  | -0.359 (p<0.05) |  |  |
| Ang-(1-7) / Ang-II |  | -0.405 (p<0.05) |  |  |
| ∑ Ang peptides |  | 0.471 (p<0.01) |  |  |
| Uv |  |  |  | 0.513 (p<0.01) |
| UvProt |  | 0.377 (p<0.05) |  |  |
